# Treatment of Dyslipidaemia in Children

**DOI:** 10.3390/biomedicines9091078

**Published:** 2021-08-24

**Authors:** Riccardo Fiorentino, Francesco Chiarelli

**Affiliations:** Department of Paediatrics, University of Chieti, 66100 Chieti, Italy; chiarelli@unich.it

**Keywords:** children, dyslipidaemia, management, non-pharmacological approach, pharmacotherapy

## Abstract

Childhood dyslipidaemia is one of the main traditional cardiovascular risk factors that initiate and exacerbate the atherosclerotic process. Healthcare providers may play a key role in the management of children with lipid abnormalities; however, they have to properly evaluate the normal lipid values and know the available treatment options in children and adolescents. Current guidelines recommend healthy behaviours as the first-line treatment for childhood dyslipidaemia. The therapeutic lifestyle changes should focus on dietary modifications, daily physical activity, reduction in body weight and tobacco smoking cessation. Parents play a key role in promoting their children’s healthy habits. In children with more severe forms of lipid abnormalities and in those who do not benefit from healthy behaviours, pharmacological therapy should be considered. Safe and effective medications are already available for children and adolescents. Statins represent the first-line pharmacological option, while ezetimibe and bile acid sequestrants are usually used as second-line drugs. Despite their limited use in children, other lipid-lowering agents (already approved for adults) are currently available or under study for certain categories of paediatric patients (e.g., familial hypercholesterolemia). Further studies are needed to evaluate the long-term efficacy, safety and tolerability of novel lipid-lowering drugs, especially in children.

## 1. Introduction

Atherosclerotic cardiovascular disease remains the leading cause of death and a major cause of morbidity [1,2]. Although clinical outcomes (e.g., death, stroke, myocardial infarction) rarely occur in youth, mounting evidence suggests that atherosclerotic lesions can begin in childhood [3].

The insidious atherosclerotic process is sped up by exposure to cardiovascular risk factors, which may be present early on in life [4]. Childhood dyslipidaemia represents one of the main traditional cardio-metabolic risk factors that exacerbate this process: lipid accumulation over time induces vascular inflammation and the development of fatty streaks, which gradually progresses to atherosclerotic plaque [5]. Interestingly, greater lipid concentrations and longer lifetime exposure to lipid abnormalities significantly correlate with the severity of atherosclerotic lesions [6].

The prevalence of children with an altered lipid profile is high: familial hypercholesterolemia (FH) affects 1 in every 250 children, while around 1 in 5 children meets the criteria for childhood overweight or obesity, which are often associated with lipid abnormalities [7,8,9]. Moreover, it is important to note that cardiovascular risk factors tend to progress from youth to adulthood [10]; several studies demonstrated that childhood lipid concentrations correlate with future adult measurements [11]. In view of this observation, it has become impossible to ignore the relevance of childhood dyslipidaemia and the need for early identification and treatment in youths with lipid abnormalities seems clear. This is very important in order to slow the atherosclerotic process and to prevent future atherosclerotic cardiovascular disease.

Given that dyslipidaemia is a modifiable cardiovascular risk factor, healthcare providers may play a key role in the management of children with lipid abnormalities. However, it is imperative to know very well the normal lipid reference values, the current recommendation and available treatment options in children and adolescents. This review aimed to outline the current status of the management of childhood dyslipidaemia, while also providing an update on the advances in the field of lipid-lowering strategies.

## 2. Pathophysiology

Dyslipidaemia is a clinical condition that is characterised by disorders of lipid metabolism. Although lipids are essential for maintaining health, abnormal lipid and lipoprotein concentrations in the blood may be dangerous. Depending on the underlying cause, childhood dyslipidaemia may be classified into primary and secondary dyslipidaemia [3]. Primary dyslipidaemia is usually caused by inherited disorders in lipid metabolism: single or multiple gene mutations (e.g., gene mutations in low-density lipoprotein receptors) may alter both lipid production and removal. Among the forms of primary dyslipidaemia, familial combined hyperlipidaemia and familial hypercholesterolemia are the most common genetic causes of dyslipidaemia. It is important to note that genetic causes are often responsible for the most severe lipid abnormalities [6]. In contrast, secondary dyslipidaemia typically occurs as the result of specific conditions, diseases or drugs that may interfere with lipid concentrations over time. The causes of secondary dyslipidaemia include obesity, diabetes, renal and chronic inflammatory diseases and corticosteroids. Secondary causes of dyslipidaemia should always be evaluated and treated; in fact, the correct management of the causative disease may often result in lipid abnormalities resolution [10].

## 3. Paediatric Guidelines

Clinical practice guidelines are designed to provide a synthesis of evidence and to translate the evidence into graded recommendations; these recommendations may be helpful in improving clinical decision making [12]. Paediatric guidelines for dyslipidaemia have undergone several modifications in recent years. The first paediatric guidelines for dyslipidaemia were published in 1992 by the National Cholesterol Education Program (NCEP) following guidelines for adults that were developed by the same NCEP [13]. Although many of the recommendations were mainly based on expert opinion rather than on systematic evidence review, these guidelines were adopted by several paediatric scientific societies. Undoubtedly, these initial guidelines engendered some controversy; however, they were important for increasing the awareness of childhood dyslipidaemia and for stimulating the research on this important topic. Moreover, the cut-off points for acceptable, borderline and high plasma lipid concentrations based on percentiles from the Lipid Research Clinical Prevalence Study were the first to be presented [14]. As new data and new evidence became available, organisations such as the American Heart Association (AHA) and the American Academy of Pediatrics (AAP) updated the original guidelines. In 1998, the AAP Committee on Nutrition produced a statement on cholesterol in childhood [15], which was followed by an additional clinical report in 2008 [16]. Furthermore, the AHA first published a consensus statement on dietary recommendations for children and then a scientific statement on drug therapy for high-risk lipid abnormalities in children and adolescents [17,18]. The most up-to-date guidelines for the management of childhood dyslipidaemia were published in 2011 by the National Heart Lung and Blood Institute (NHLBI) after performing a systematic review and grading the best available evidence [3]. The 2011 Guidelines constitute a part of an integrated approach with a focus on all cardiovascular risk factors in children and adolescents; they represent a cornerstone for cardio-metabolic risk reduction and cardiovascular health in youth. As regards lipid abnormalities, the NHLBI Guidelines outlined the currently used references values for plasma lipid, lipoprotein and apolipoprotein concentrations in children and adolescents (Table 1); moreover, they give recommendations concerning both lipid assessments in youth and the management of paediatric lipid disorders.

In accordance with previous guidelines, the non-pharmacological approach (including dietary and lifestyle modifications) remained an integral part of the treatment for dyslipidaemia in children and adolescents; on the other hand, the recommendations for pharmacotherapy substantially changed in comparison with previous guidelines [19]. Due to the increasing data on efficacy and safety, along with the Food and Drug Administration (FDA) approval for the use of several lipids lowering drugs in children, the therapeutic options for children with dyslipidaemia have expanded and, nowadays, statins are the preferred drugs in paediatrics [3,20].

## 4. Management of Dyslipidaemia in Children

### 4.1. Non-Pharmacological Approaches

Although several pharmacological treatments are available or under development, current guidelines recommend healthy behaviours as the first-line treatment for childhood dyslipidaemia. It is important that healthy behaviours should be recommended for all children and adolescents; however, they should be strongly encouraged in those children with borderline or high plasma lipid and lipoprotein concentrations.

For children with an altered lipid profile, the initial management should consist of therapeutic lifestyle changes that focus on dietary modifications, daily physical activity, improving body weight and tobacco smoking cessation [21]. Moreover, in order to prevent obesity in children, they should be encouraged to sleep for a decent amount of hours per day and to limit screen time (including television, cell phone, computer use, videogames, handheld electronics) to less than 2 h per day. In 2016, The AAP consensus groups recommended adequate daily hours of sleep (including naps) for children and adolescents, depending on their age [22]. As regards sedentary activities, in a recent study, it was observed that every additional hour of watching television was correlated with increased triglycerides (TG) and decreased high-density lipoprotein (HDL-C) levels [23]. Several possible reasons were proposed: among them, the lower energy expenditure and the increased intake of energy-dense foods (e.g., soft drinks, fast food) while watching television.

Although dietary treatment remains under debate, a modified diet can improve abnormal lipid profiles by inducing a lipid-lowering effect, mainly on triglycerides (TG) levels, but it also has a modest impact on total cholesterol (TC) and low-density lipoprotein (LDL-C). In adults, the PREDIMED study (the largest dietary prevention trial) demonstrated that the Mediterranean diet is beneficial in reducing the incidence of major cardiovascular events. Similarly, adherence to the Mediterranean diet in children may improve the carotid intima-media thickness test (CIMT), which is an early marker of atherosclerosis [24,25]. In view of these observations, it is likely that dietary modifications are relevant for the prevention of atherosclerotic cardiovascular disease in both children and adults. The specific dietary changes should emphasise decreasing total, trans and saturated fats; decreasing cholesterol amounts; and increasing the intake of fibre. The NCEP suggests two approaches for proposing a modified diet: the population approach is a group of recommendations for all youths in order to prevent an abnormal lipid profile and the atherosclerotic process. In contrast, the individual approach consists of suggestions for children with confirmed dyslipidaemia and an increased risk for cardiovascular disease. It is important to note that this latter approach uses a two-step nutritional change (CHILD-1 and CHILD-2) and that CHILD-1 recommendations coincide with those of the population approach [26]. The recommended population diet, as well as the diet Cardiovascular Health Integrated Lifestyle Diet-1 (CHILD-1), should limit the total fat consumption to 20–30% of total calories, saturated fat intake to less than 10% of total calories and average cholesterol ingestion to less than 300 mg/day. Children should also avoid trans-fatty acids (<1%), preferring polyunsaturated fatty acids and monounsaturated fatty acids, which should be up to 10% and between 10 and 15% of total daily calories, respectively. It is also recommended to increase the intake of dietary fibre through whole grains, vegetables and fruit (five or more a day). For children at an increased cardiovascular risk and for children with confirmed dyslipidaemia who have failed to achieve the lipid goals after 3 months of the CHILD-1 diet, more intensive restrictions are needed. The Cardiovascular Health Integrated Lifestyle Diet-2 (CHILD-2) requires limiting saturated fat intake to less than 7% and cholesterol average ingestion to less than 200 mg/day [3,27]. The other step 1 recommendations should not be interrupted through the step 2 diet.

It is noteworthy that the NCEP recommends dietary modifications in children from 2 years of age: the first two years of life are critical for the development and the growth of children and it is important to provide them with an adequate amount of calories and nutrients [28]. This also applies to children older than 2 years old and both CHILD-1 and CHILD-2 should ensure adequate daily caloric intake for normal growth and development: as a consequence, these diets should consist of 50–60% of total daily calories from carbohydrates and 10–20% from proteins [27]. It is never suggested to limit protein consumption, while in children with elevated TG, it is recommended to decrease simple sugar consumption (including fruit juices and sugar drinks) and replace them with complex carbohydrates [28].

For children with hypertriglyceridemia, an increase in omega-3 fatty acid dietary intake should also be encouraged by increasing the consumption of fish. Long-chain omega-3 fatty acids are also available in the form of unregulated fish oil products and prescription drugs; although it is not clear the exact mechanisms by which they reduce TG concentrations and limited data exist in children and adolescents, prescription products seem to lower TG levels and have been safely used in children [20,29,30]. However, they lack the approval for use in children and should be used in consultation with a lipid specialist. For patients with increased LDL-C values, the CHILD-2 diet also recommends dietary adjuncts, such as plant stanol and sterol esters and water-soluble fibre psyllium. Plant sterol and stanol, when taken up to 2 g per day, were shown to inhibit intestinal cholesterol absorption, leading to a reduction in LDL-C levels by approximately 9% [31].

Although the effectiveness of dietary changes is variable, it is important to remember that the above-mentioned dietary modifications are safe and well tolerated over time. Several studies, such as the Special Turku Coronary Risk Factor Intervention Project (STRIP) and the Dietary Intervention Study in Children (DISC) showed that reducing fat intake (total fat, saturated fat and cholesterol) was not significantly associated with changes in somatic growth, pubertal development, mean body mass index, nutritional sufficiency and psychological/social features [32,33]. Moreover, a recent study concluded that beneficial nutritional interventions can be safely introduced in youth and sustained over 20 years [34].

Consultation with a registered nutritionist may help with promoting long-term adherence to a diet; a study of 1062 children (540 children in the intervention group and 522 controls) showed that repeated dietary counselling was helpful in reducing both saturated fat consumption and LDL-C concentrations [35]. In addition, a paediatric dietitian plays a key role in setting goals, tracking progress, making dietary adjustments and educating parents about nutritional plans inside and outside of the home [21]. It is important to set realistic short-term dietary goals and to consider social, parental and cultural factors in order to ensure the effective implementation of nutritional changes [36]. It is also important that dietary modifications should never be portrayed as punitive, but rather in terms of the child’s education, and that adequate non-food-based rewards should be given when accomplishing the goals. Improving the quantity and the quality of nutrition is equally important: children with obesity often consume exaggerated portions and large quantities of non-nutritive but calorie-dense food. Common sources of non-nutritive foods include ultra-processed products, soft and energy drinks, snacks and fast food [27]. The elimination of these foods and limitation of portion sizes should be strongly encouraged to improve the nutritional status. Furthermore, it is crucial that children and adolescents avoid skipping meals (in particular breakfast); in accordance with a retrospective, observational study, children who consumed fewer than two meals per day had higher levels of TC and LDL-C compared with those eating three times or more per day [37].

In addition to nutritional changes, physical activity and weight reduction are the cornerstones of preventing and treating lipid abnormalities in children. Physical activity is associated with a variety of health benefits, both in healthy children and in youths with chronic disease [38]. The benefits of physical activity were widely documented and include improved musculoskeletal, mental, behavioural and cardiovascular health. In particular, being physically active has a positive effect on cardiorespiratory fitness, serum glucose concentrations and insulin sensitivity, blood pressure, bone density and lipid profile [39]. As a consequence, regular physical activity should always be encouraged in children with dyslipidaemia; it may be useful in lowering TC, TG and LDL-C levels, increasing HDL-C and, more importantly, it may assist with body fat and body mass index (BMI) reduction. Therefore, it is critical that all children and adolescents should engage in at least 1 h of moderate-to-vigorous physical activity every day [3]. Interestingly, a study of 1235 adolescents showed a dose–response relationship between an increased number of minutes of physical activity and improved lipid concentrations (HDL-C and TG values) [40]. It is important that physical activity is age appropriate, various (including unstructured and structured activities) and enjoyable to the child. Further recommendations are available in the 2018 Physical Activity Guidelines; these guidelines, released by the US Department of Health and Human Services, provide important guidance on the amounts and types of physical activity for multiple paediatric populations groups [41].

Weight management is another important recommendation for children with an altered lipid profile and represents the primary treatment goal for obese or overweight children with dyslipidaemia. The excess adiposity adversely affects not only the lipid profiles but the entire cardio-metabolic health of young people [42]; it is, therefore, necessary to maintain a healthy BMI. A 5 to 10% reduction in body weight through dietary modifications and increased physical activity is beneficial for reducing cardiovascular risk and improving lipid abnormalities. Via different mechanisms (improved insulin sensitivity, enhanced activity of lipoprotein lipase, reduced free fatty acids release from adipose tissues), weight loss is expected to increase the TG catabolism and removal by approximately 20% [43,44]. Only when obesity-related comorbidities (such as dyslipidaemia) are not sufficiently reduced with adequate weight reduction, they should be treated independently [6].

### 4.2. Family-Based Approach

Parents play a key role in promoting healthier eating habits and adequate activity levels in their children and several authors consider parents one of the main focuses of childhood dyslipidaemia prevention and treatment [45]. This is a critical point, although it is often overlooked. Therapeutic lifestyle changes should be adopted by the entire family: if the whole family does not change their habits, dyslipidaemia is unlikely to improve [28]. First, parents are responsible for the portions served to the child and for food and beverages that enter the home. It is important to note that the food available in the home is often the food that children learn to consume and that children’s consumption of fruit and vegetables is predicted by their availability [46]. Second, parents play a role in creating a healthy home environment (e.g., a smoke-free environment) and promoting healthy habits (e.g., a healthy sleep routine); important factors that may affect children’s lipid profile are the sources of food (prepackaged or homemade meals), TV watching during the meal and the frequency of family meals [47]; the promotion of regular family meals is protective for obesity and its related consequences, such as dyslipidaemia [48]. Third, considering that children often mimic their close family members, parents serve as models for healthy behaviours and are very important for children’s education in terms of the amount of physical activity and eating habits [49]. In view of this, and considering that the introduction of healthy behaviours at a young age may be carried throughout adult life, families must promote a healthy lifestyle.

### 4.3. Pharmacological Treatment

Although secondary dyslipidaemia (e.g., obesity-related dyslipidaemia) is usually successfully treated with the management of the underlying disorder and therapeutic lifestyle changes, the adoption of healthy habits and long-term adherence to lifestyle modifications may be challenging. Moreover, children with primary dyslipidaemia (e.g., FH) do not equally benefit from healthy behaviours: in children with FH, a low-fat diet was shown to have only modest effects, leading to an LDL-C reduction by approximately 10% [50,51]. In such cases, as well as in children with more severe forms of lipid abnormalities, the use of pharmacological therapy should be considered. This could be possible thanks to the approval in children of safe and efficient pharmacological options. According to the NHLBI Guidelines, decisions regarding the need for drug treatment should take account of the baseline lipid levels, the child’s age, the presence of moderate-to-high cardiovascular risk factors or condition and the familial history of premature cardiovascular disease [3]. In particular, the NHLBI Guidelines state that children with LDL-C > 250 mg/dL or TG > 500 mg/dL should consult a lipid specialist in order to promptly start the pharmacological treatment. In children with less severe lipid abnormalities, medication therapy should be recommended after at least 6 months of therapeutic lifestyle changes if LDL-C > 130–190 mg/dL; the LDL-C threshold at which statin therapy should be initiated may depend on the number of cardiovascular risk factors or condition and the familial history of premature cardiovascular disease. Basically, the goal of pharmacological therapy in children is to obtain acceptable values of LDL-C (<130 mg/dL); however, children with high-risk cardiovascular conditions (e.g., FH), may require stricter LDL-C targets (<100 mg/dL) [3,6]. It is important that healthy behaviours should be implemented even though medications are initiated: therapeutic lifestyle changes may be useful as a synergic mechanism to improve the lipid profile and for the lowering dosages of lipid-lowering drugs [52].

#### 4.3.1. Approved Pharmacological Treatment for Children

##### Statins

At present, statins have supplanted bile acid sequestrants as the first-line pharmacological therapy for children with dyslipidaemia. By inhibiting 3-hydroxy-3-methylglutaryl-CoA reductase (HMG-CoA reductase, which is the rate-limiting enzyme of the hepatic cholesterol production), statins reduce the intracellular cholesterol amount, which leads to the upregulation of the LDL-C receptor on the hepatocyte’s surface and increases LDL-C catabolism [53]. In adults, it is well known that statins are safe and effective at reducing cardiovascular morbidity and mortality in both primary and secondary prevention [54]. As with adults, there is growing evidence that statin therapy is safe, well tolerated and efficient at lowering lipids levels in children and adolescents with dyslipidaemia. Over time, several studies were published on this issue [55,56,57,58,59]. A recent meta-analysis showed that statins were effective at reducing TC, LDL-C and TG by approximately 25%, 33% and 8%, respectively; moreover, they led to a mean relative increase in HDL-C of approximately 3%. Although the available statins had a variable lipid-lowering efficacy throughout the trials (being rosuvastatin and atorvastatin the most potent statins), the results indicated a dose-dependent effect [58]. In the study of Luirink et al., it was also demonstrated that statins were helpful in slowing the progression of CIMT and reducing cardiovascular morbidity and mortality when used early in children with FH. Interestingly, the beneficial effect was present even though the lipid target goals were not achieved [59]. As regards the safety profile, no significant differences in short and long-term adverse events were described when statins were compared to a placebo. In a 10-year follow-up study, Kusters et al. did not note any differences in growth, maturation (including sexual development, as assessed by Tanner staging) or educational level between treatment and placebo groups [56]; in a study in which a 20-year follow-up was performed, no differences in transaminases (over 3-fold increase) and creatine kinase (over 10-fold increase) levels or serious adverse events were described between statin therapy and placebo [59]. It is interesting to note that only 2% of children permanently discontinued the treatment due to adverse events and more than 80% of the youths were regularly using statins [59]. These results suggest that statins are generally well tolerated and that patients have good long-term compliance.

There are currently seven approved statins for use in children and adolescents: lovastatin (the first HMG-CoA reductase inhibitor), simvastatin, atorvastatin and fluvastatin are indicated for children and adolescents ≥10 years old; pitavastatin and pravastatin have been approved for use in children from 8 years old; rosuvastatin is indicated in children as young as 6 years old [53]. The choice of the statin can be influenced by healthcare provider preferences, baseline LDL-C concentrations, treatment goals and expected LDL-C reduction with a particular formulation [10]. When using a statin, it is recommended to start with the lowest available dose once daily (at bedtime) and subsequently increase the dose up to the maximally tolerated/age-appropriate dose, if necessary [3]. It is also advised to increase the dose by one increment (doubling the dose), without adjusting the dose based on body weight.

All statins, available at varying dosages, were approved for children with FH. Several scientific societies provided recommendations regarding statin treatments for children with both heterozygous FH (HeFH) and homozygous FH (HoFH) [3,6,60,61,62]. Depending on the guidelines we use, children from 6-10 years old with HeFH are potential candidates for pharmacological treatment in order to achieve LDL-C values < 100–130 mg/dL or a 50% reduction in LDL-C from baseline concentrations. As regards children with HoFH, the common denominator that guidelines share is that statin treatment should be started at the time of diagnosis, regardless of age. When used early, statins considerably reduce the cardiovascular risk of HoFH patients, changing the natural history of the disease and improving the prognosis. Although statins alone are rarely sufficient for achieving the LDL-C goals, they represent the pillar of the treatment of HoFH patients [53].

Although it was shown that statins are safe, all children and adolescents on statin treatment should always be monitored: growth, maturation, pubertal development and possible adverse effects should be regularly assessed. In addition, baseline transaminases and creatine kinase levels should be evaluated before starting the treatment and then repeated over time (when the dose of statin is modified, muscle symptoms occur and at regular intervals). If symptoms or laboratory abnormalities are reported, statin treatment should be temporary interrupted and restarted after the resolution. For those patients that are intolerant to statins, it is possible to try another statin formulation before using a non-statin pharmacological treatment [63].

As a major substrate of cytochrome P450, it is important to know that statin therapy is associated with various drug interactions; macrolides and antifungal azoles, which are commonly prescribed to children, are metabolised by the same enzyme and may be responsible for increasing serum statin levels and its associated adverse effects [19]. Even in children with chronic kidney diseases, statins should be used carefully: dose adjustment is needed and only simvastatin and atorvastatin can be prescribed, as they are only metabolised in the liver [64]. In addition, statins are contraindicated in pregnancy and lactation due to their possible teratogenicity: as a consequence, all post-pubertal girls on statin treatment should be adequately counselled and receive appropriate contraception [65].

##### Ezetimibe and Bile Acid Sequestrants

Although a majority of children usually benefit from statin treatment, some children with the most severe lipid abnormalities (e.g., HoFH) may require other lipid-lowering drugs in order to achieve the LDL-C therapeutic target [66]. This also applies to those rare patients who develop an intolerance to HMG-CoA reductase inhibitor. If the LDL-C goals are not attained, before adding second-line therapeutic drugs, it is possible to increase the dose of statin or switch to a more potent statin, such as rosuvastatin or atorvastatin [53]. Moreover, it is extremely important to closely monitor the adherence to treatment among patients with a poor response [67].

At present, bile-acid-binding resins (or sequestrants) and ezetimibe are the only approved non-statin treatment for children and adolescents.

Due to its inhibition of intestinal cholesterol absorption (blocking the Niemann-Pick C1-Like Intracellular Cholesterol Transporter 1), ezetimibe is the most frequently used second-line agent [68]. It may be used as a monotherapy or combined with statins if the maximal dose of statins is insufficient for achieving the LDL-C target. A 12-week trial of ezetimibe as a monotherapy showed a significant reduction in LDL-C (by 27%) and TC (by 21%) when compared to a placebo [69]. As regards the statin–ezetimibe association, another long-term trial showed that the combination treatment led to a greater reduction in LDL-C by 10–15% compared to a statin monotherapy [70]. In children and adolescents with HeFH, the statin–ezetimibe association makes it possible to achieve the LDL-C goal in approximately 69% of children [71]. Although data in children is limited, ezetimibe is generally safe and well tolerated: no clinically significant adverse events, laboratory abnormalities (transaminases and muscular creatine kinase increase) nor impacts on growth and maturation have been associated with ezetimibe. Transient diarrhoea is probably the most common adverse effect of ezetimibe [52,69,70,71]. Ezetimibe is currently approved for children with FH from 10 years of age; the licensed formulation is 10 mg. Contrary to statins, which are usually taken at bedtime, ezetimibe should be administrated without regard to time of day or meals.

Bile acid sequestrants, such as cholestyramine and colesevelam, represent additional lipid-lowering drugs. As mentioned above, they were the only pharmacological treatment recommended by the first paediatric guidelines; however, they are no longer a first-line therapy [13]. Bile acid sequestrants, while not being adsorbed, are able to bind to bile salt in the gut and decrease intestinal cholesterol absorption. This results in greater hepatic conversion of cholesterol to bile, upregulation of the LDL-C receptor and a consequent reduction in TC and LDL-C concentrations. Their efficacy on lipid profiles varied between studies. Cholestyramine (when taken at 8 g per day) reduces LDL-C values by approximately 15%; colesevelam (3.75 g/day) has a similar effect, reducing LDL-C by 12–13% [72,73]. Despite the additive lipid-lowering effect of bile acid sequestrants upon statins, these drugs are not frequently used. Cholestyramine, in particular, is associated with poor palatability and gastrointestinal effects (diarrhoea, nausea, abdominal pain, vomiting), and poor tolerance as a consequence [53]. It is also important to note that, contrary to ezetimibe, bile acid sequestrants may also affect the intestinal absorption of fat-soluble vitamins, such as vitamin D [73]. In view of this, they are generally used as additional agents in children with an LDL-C that has not reached the target level despite the optimised statin–ezetimibe combination [3]. Colesevelam is the only bile acid sequestrant that is approved by the FDA for FH children over 10 years of age.

#### 4.3.2. Non-Approved Pharmacological Treatment for Children

##### Fibrates and Niacin

Because limited data exist on the use of niacin and fibrates in children, these lipid-lowering drugs lack FDA approval. According to Colletti et al., niacin (or vitamin B3) may be helpful for reducing LDL-C and TC and increasing HDL-C values; this is mainly due to the reduced hepatic production of very low-density lipoprotein (VLDL-C) [74]. However, the tolerance is poor and side-effects may be very common and serious (including headache, flushing, abdominal pain, liver failure, myopathy, impaired glucose tolerance). As a consequence, the use of niacin is usually reserved as an adjunct option for children with severe dyslipidaemia (e.g., HoFH) that are not achieving the LDL-C goals [51].

Fibrates work by activating the transcription factor named peroxisome proliferator-activated receptor alpha (PPARα), which plays a key role in regulating hepatic lipid metabolism. Via different mechanisms, fibrates lower the hepatic TG production, inhibit the production of VLDL-C and reduce peripheral lipolysis [20]. Although they have variable effects on LDL-C, fibrates may be beneficial in reducing TG in children and adolescents. In particular, they are often the first-line treatment in children with severe TG abnormalities (e.g., primary hypertriglyceridemia) in order to reduce the risk of pancreatitis. Depending on the baseline TG concentrations, fibrates are able to reduce serum TG by 40–60% [10]. The adverse effects that are associated with fibrate treatment are similar to those with statins.

Due to the limited experience in children and the insufficient evidence on its safety and dosage, it is recommended that niacin and fibrates should be initiated by a lipid specialist.

#### 4.3.3. Additional Lipid-Lowering Options

In children with more severe forms of dyslipidaemia, such as HoFH, given the relevant cardiovascular morbidity and mortality (the age of death is approximately 18 years in untreated patients with HoFH), it is extremely important to keep lipid concentrations in range, whenever possible [52]. In order to achieve this goal, as mentioned above, the prompt detection of the disease represents a cornerstone of the treatment, as well as the early start of lipid-lowering drugs (at diagnosis) [53]. However, children with HoFH respond poorly to conventional treatment despite receiving the maximally tolerated statin dose or combination therapy with bile acid sequestrants and/or ezetimibe [75]. Therefore, additional therapeutic options may be considered.

Lipoprotein apheresis represents an additional option that is recommended for children with HoFH. Even though it is expensive and difficult in practice, weekly or bi-weekly LDL-C apheresis is effective at lowering LDL-C levels by 50–70%, improving cardiovascular health and providing clinical benefits (regression of xanthomas) [64]. Nevertheless, it is important to note that the effect on LDL-C concentrations is temporary and the desired frequency of lipoprotein apheresis should be every 1–2 weeks [76]. Lipoprotein apheresis should be started as early as possible; however, being an invasive procedure, it cannot always be performed due to several difficulties. Therefore, it should be considered from the age of 5 years old and initiated before the age of 8 years [77]. In a recent study, it was found that LDL-C apheresis is safe and is mainly associated with minor side effects; possible adverse events include abdominal pain, hypotension and iron deficiency [64,78].

More recently, novel lipid-lowering drugs were developed and others are in the process of human experimentation (Table 2).

Both evolocumab and alirocumab are direct monoclonal antibodies that target the proprotein convertase subtilisin/kexin type 9 (PCSK9). By inhibiting PCSK9, they prevent the degradation of LDL-C receptors and favour its recycling on the hepatocyte surface, resulting in greater expression of LDL-C receptors on the cell membrane and improved LDL-C catabolism [96]. In adults, evolocumab and alirocumab were helpful in reducing LDL-C by approximately 20–25% and 55–60% in patients with HoFH and severe HeFH, respectively [79,80,82,83,97]. As regards children, evolocumab was used with HoFH and HeFH before the age of 18 years and was shown to be as well tolerated and effective as in adults [79,81]; similarly, when initiated in children with HeFH, alirocumab demonstrated significant reductions in LDL-C and a good safety profile [84]. PCSK9 inhibitors are very promising and should be considered in children from the age of 12 years old who are intolerant to statins or not responding to conventional treatment; however, the presence of the null mutation of LDL-C receptors may limit their utility [98]. A similar approach may consist of the use of inclisiran, which is a gene-silencing drug that inhibits the hepatic production of PCSK9 through a small interference RNA [53]. In adults, it seems to be effective, safe and well tolerated thanks to the possibility of being administered at long time intervals [85]. For paediatric patients, inclisiran is still under evaluation.

Evinacumab is a monoclonal antibody that binds and inhibits the function of angiopoietin-like 3 (ANGPTL3). ANGPTL3 plays a key regulatory role in lipid metabolism and is an inhibitor of lipoprotein-lipase. ANGPTL3 deficiency is traditionally associated with reduced cardiovascular risk and serum lipid concentrations; as a consequence, the inhibition of ANGPTL3 by evinacumab may be a new therapeutic option for the management of dyslipidaemia [99]. In adults with HoFH, evinacumab has documented potential benefits, leading to an average reduction in LDL-C by 47%. Interestingly, evanicumab was effective regardless of the presence of a null variant of LDL-C receptors [86]. Although it presented similar adverse effects to placebo in adults, further investigations are needed in children and adolescents. It was recently approved by the FDA as an add-on treatment for patients with HoFH from the age of 12 years old. Novel pharmacological options include mipomersen, lomitapide, bempedoic acid and anacetrapib. Mipomersen is an anti-sense oligonucleotide that is responsible for the degradation of mRNA translation in apolipoprotein B (APO-B). This drug inhibits the hepatic production of APO-B and reduces the assemblage of VLDL-C and other atherogenic lipoproteins [87]. Mipomersen is associated with an average reduction in LDL-C of 28% from baseline in addition to conventional therapy (e.g., maximum dose of statin) and is currently approved by the FDA for patients with HoFH and severe HeFH from the age of 12 years [64,87]. In children with HoFH, it was shown to be effective in reducing LDL-C but was poorly tolerated due to side effects, such as flu-like symptoms and reactions at the injection site [88].

On the other hand, lomitapide is a microsomal transfer protein of triglycerides (MTTP) inhibitor; given that MTTP mainly acts by binding TG to APO-B in the liver, lomitapide interferes with VLDL-C assembly and production [87]. Although adverse effects (e.g., gastrointestinal symptoms and fatty liver) occur frequently, lomitapide may lead to significant reductions in VLDL-C, LDL-C and TG by approximately 65%, 51% and 56%, respectively [89]. At present, it is only approved for adult patients with HoFH; however, it has been used on a compassionate basis in few paediatric patients. In children with HoFH, the administration of lomitapide reduced the LDL-C values by 58.4% and target LDL-C goals were achieved in more than half of the patients [90,91].

Bempedoic acid is a targeted therapy that is designed to reduce cholesterol biosynthesis, by inhibiting the adenosine triphosphate-citrate-lyase (ACL). In adults with FH, it is approved as a monotherapy or in combination with ezetimibe, in addition to diet and statin therapy [100]. When used as a monotherapy, it may further reduce LDL-C by 17% [92]; the combination of ezetimibe–bempedoic acid makes an additional reduction in LDL-C by 38% possible [93]. Trials using children are not yet available. Lastly, anacetrapib and other cholesterol ester transfer protein (CEPT) inhibitors may be a useful tool for the management of dyslipidaemia in the future. By reducing the movement of cholesterol esters from HDL-C to VLDL-C and LDL-C in exchange for TG, CEPT inhibitors significantly increase HDL-C and reduce VLDL, LDL-C and TG. Anacetrapib was shown to be responsible for an increase in HDL-C by 140% and a decrease in LDL-C by 40%. It is interesting to note that anacetrapib is also associated with improved glucose homeostasis and decreased atherosclerotic cardiovascular events [94,95]. Although CEPT inhibitors are not approved by the FDA in children or adults, the potential utility of anacetrapib and the emerging data from genomic analyses may suggest their potential role in clinical practice [101].

## 5. Conclusions

**Figure 1:**
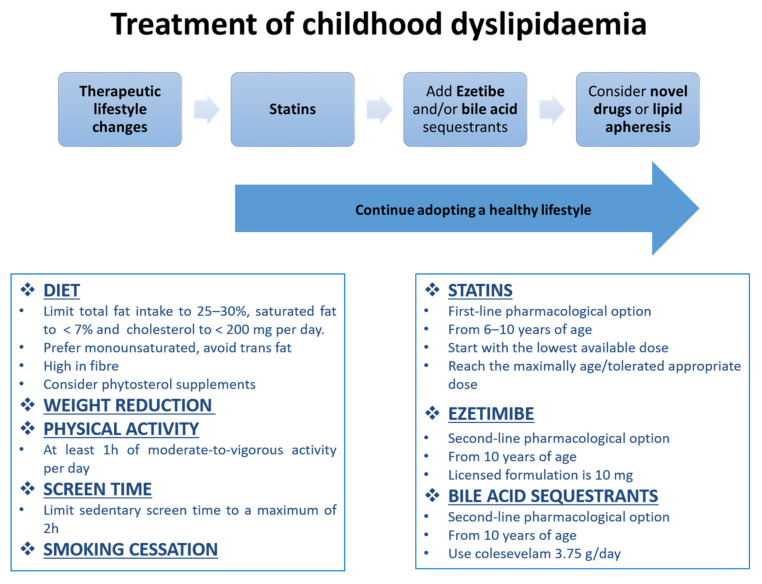
Treatment algorithm of childhood dyslipidaemia.

An altered lipid profile over time plays a key role in the initiation and progression of atherosclerotic process in children and adolescents. Given that atherosclerosis is a “paediatric problem” that correlates with future cardiovascular health, it seems necessary to recognise it early and promptly treat children with modifiable cardiovascular risk factors, such as dyslipidaemia. It is important to act when atherosclerosis is reversible. Several guidelines and recommendations were published regarding the management of lipid abnormalities in children; however, knowledge and uptake of these guidelines and recommendations are not optimal in both patients and clinical practitioners. It is therefore important to promote and increase awareness of them in this field. A graduated, individualised and multidisciplinary approach is the basis of the treatment of dyslipidaemia: therapeutic programs should combine behavioural modifications, dietary changes, physical activity, drugs and other measures. Several figures may be involved in the management of children with dyslipidaemia: parents, personal trainers, nutritionists, clinical practitioners, lipid specialists and the patients themselves; among them, the family plays a very important role in educating the children and creating a healthy environment. Although there can be difficulties in adopting and sustaining therapeutic lifestyle changes, they represent the cornerstone of the treatment and should be adopted all the time, regardless of drug therapies. As regards pharmacological treatment, approved safe and effective medications are already available for children and adolescents. As a consequence, physicians and patients should not hesitate to consider lipid-lowering drugs in children, where statins represent the first-line option; there is growing evidence that statin therapy in children may change the natural history of atherosclerosis, even if lipid target goals are not achieved. In addition to statins, there are other therapeutic strategies. Figure 1 illustrates the algorithm for the treatment of childhood dyslipidaemia.

Out of the traditional options (ezetimibe, bile acid sequestrants, fibrates, niacin, LDL-C apheresis), additional lipid-lowering agents are currently available or under study for certain categories of patients (e.g., FH). At present, the prescription of novel lipid-lowering drugs beyond their licence should be considered on a case-by-case basis for the most severe lipid abnormalities or for preventing risky invasive procedures (e.g., lipid apheresis). A multidisciplinary team that includes lipid specialists should be involved. In the future, novel lipid-lowering drugs may be promising options for the management of paediatric dyslipidaemia; however, further studies are needed to evaluate their long-term efficacy, safety and tolerability.

## Figures and Tables

**Table 1:** Lipid and lipoprotein reference values and corresponding centile in children and adolescents.

		ACCEPTABLE		BORDERLINE		HIGH	
		mg/dL	Percentile	mg/dL	Percentile	mg/dL	Percentile
Children and Adolescents	TC	<170	<75th	170–199	75–95th	>200	>95th
	LDL-C	<110	<75th	110–129	75–95th	>130	>95th
	TG						
	0–9 years	<75	<75th	75–99	75–95th	>100	>95th
	10–19 years	<90	<75th	90–129	75–95th	>130	>95th
	HDL-C	>45	>10th	40–45		<40	<10th
	Non-HDL-C	<120	<75th	120–144	75–95th	>145	>95th

Adapted from the Expert Panel on Integrated Guidelines for Cardiovascular Health and Risk Reduction in Children and Adolescents.

**Table 2:** Novel lipid-lowering drugs.

	Mechanism of Action	Mean LDL-C % Reduction	Currently Approval Status	Trials in Children	Studies
Evolocumab	PCSK9 inhibitor	20–55%	FH from 12 years of age	HoFH or severe HeFH from 12 years of age	Santos et al. [79] Hovingh et al. [80] Santos et al. [81]
Alirocumab	PCSK9 inhibitor	21–62%	FH in adults	Severe HeFH from 8 years of age	Blom et al. [82] Hartgers et al. [83] Daniels et al. [84]
Inclisiran	Small interfering RNA directed against PCSK9	35–43%	Primary hypercholesterolemia or mixed dyslipidaemia in adults	Will be tested in paediatric HeFH in the ORION-16 study.	Raal et al. [85]
Evinacumab	ANGPTL3 inhibitor	42–51%	HoFH from 12 years of age	HoFH from 12 years old	Raal et al. [86]
Mipomersen	Antisense APO-B synthesis inhibitor	28%	FH from 12 years of age	HoFH and severe HeFH from 12 years of age	Blom et al. [87] Raal et al. [88]
Lomitapide	MTTP inhibitor	51%	FH in adults	HoFH from 7 years of age	Stefanutti [89] Ben-Omran et al. [90] Chacra et al. [91]
Bempedoic acid	ACL inhibitor	17–38%	Primary hypercholesterolemia or mixed dyslipidaemia in adults		Goldberg et al. [92] Ballantyne et al. [93]
Anacetrapib	CEPT inhibitor	40%	Not approved		Filippatos et al. [94] Armitage et al. [95]

## Data Availability

Not applicable.
